# Perceptions of Stakeholders Regarding China’s Special Education and Inclusive Education Legislation, Law, and Policy: Implications for Student Wellbeing and Mental Health

**DOI:** 10.3390/bs13060515

**Published:** 2023-06-19

**Authors:** Ahmed Alduais, Meng Deng, Hind Alfadda

**Affiliations:** 1 Department of Human Sciences, University of Verona, 37129 Verona, Italy; 2 Department of English and Communication, The Hong Kong Polytechnic University, Hong Kong, China; 3 Department of Special Education, East China Normal University, Shanghai 200062, China; mdeng@ed.ecnu.edu.cn; 4 Department of Curriculum and Instruction, King Saud University, Riyadh 11362, Saudi Arabia

**Keywords:** special education, inclusive education, policy, law, legislation, China

## Abstract

Laws and policies, no matter how well designed, can fail if they are not implemented correctly. This can occur when there is no interaction between policymakers and those who are working on the ground. The purpose of this study was to determine the understanding of Chinese stakeholders regarding legislation, policy, and law associated with the provision of special education and explore its implications for student wellbeing and mental health. Two questions were posed: (1) Does a stakeholder’s attitude towards legislation, policy, and law regarding special education impact their role or responsibilities? (2) In what ways do stakeholders interact with legislation, laws, and policies regarding special education and their work experience in the field? Using in-depth interviews as the basis for the study, researchers gained valuable insight into how administrators, practitioners, and academics perceive laws and policies. Participants exhibited exaggerated attitudes and over-interpretations of some of these items, which we attribute to partly real factors, as well as nationalistic or patriotic feelings. The evidence included calls for specific laws and policies as well as a switch from a top-down to a bottom-up approach to reform to bridge the disparity between different regions in the country. As the participants agreed, there have also been remarkable achievements in building a more comprehensive and inclusive system over the last decade. However, the gaps between rural and urban areas, primary and middle schools, high schools and vocational schools need to be addressed urgently in specific laws and policies. Addressing these disparities will not only improve the overall quality of special education but also have significant implications for student wellbeing and mental health. By ensuring that all students have access to tailored support and resources, policymakers can foster a more inclusive and supportive environment that promotes positive mental health outcomes for all learners.

## 1. Introduction

### 1.1. Law, Legislation and Policy

For any policy to be successfully implemented, the interaction between policies, policy-makers, decision-makers, and stakeholders is essential. In the context of special education and inclusive education in China, it is worthwhile to explore the perspectives and experiences of administrators, practitioners, and academics in the field in relation to legislation, law, and policy. 

To gain a better understanding of the status of special and inclusive education policies, we present the following synthesis. Fu et al. mentioned that “in several key government documents on national educational development, the Chinese government persists in developing more special schools” [1] (p. 581) but also raised their concern on “the contradictions in the new regulations reflecting long-rooted disagreements about inclusive education in reality” [1] (p. 581). Further, barriers hindering the development of inclusive education include a competitive exam-based school system, large class sizes (≥45), economic development and more investment, three modes of education (i.e., learning in regular classrooms (LRC), mainstreaming, and home learning), and a paucity of inclusive education in senior middle school (i.e., high school). In order to develop inclusive education, the authors proposed: improving legislation for inclusive education in general, providing detailed laws and policies, identifying the responsible agencies and roles of each in relation to special education and inclusive education, involving the community in legislation processes, repealing laws that are not consistent with the Convention on the Rights of Persons with Disabilities (CRPD), for instance, the law which requires opening special education schools in counties with populations of more than 300,000, as this encouraged segregation and decreased the expansion of inclusion [1].

Moreover, Huang and Zhang conducted a study on the allocation of special education resources in 31 provinces of China (excluding Macau, Hong Kong, and Taiwan) with reference to the National medium and long-term educational reform and development program (2010–2020) and the thirteenth 5 Year Plan for National Education Development [2]. The authors found “Shanghai, Liaoning, Shandong, Jilin, Tianjin, Hebei, Zhejiang, Heilongjiang, Shanxi, and Beijing” are the top provinces in resources allocation of special education as compared to the lowest ones “Sichuan, Chongqing, Fujian, Jiangxi, Guizhou, Guangxi, Qinghai, Yunnan, Hainan, and Anhui”, with the rest 11 provinces in the middle [2] (p. 19). The authors also mentioned that while both Beijing and Shanghai have the advantage of being the most developed provinces, they have the disadvantage of having the largest number of special education students [2]. The authors recommended the issuance of province-based policies and the allocation of resources to bridge the gap in the imbalance among the provinces and provide equal education to people with disabilities, increasing the number of special education schools, the recruitment and preparation of teachers that are more professional, communication among provinces, research on special education and increasing numbers of LRC students [2]. 

Based on the description by authors in reference [2], the first indication of special education appeared in the Book of Rites (i.e., Chinese classics), influenced by Confucian thoughts. Among these is the idea that there must be social harmony, albeit it looked at people emotionally with the view that their disability is due to evil power and ranking them at the lowest social level. The second indicator is the Western missionaries in the mid-19th century, followed by the open reform in 1986. The open reform resulted in major reforms in special education, including the Compulsory Education Law in 1986, moving to inclusive education (i.e., LRC) between 1980 and 1994, the National Plan 2010–2020, and the thirteenth 5 year education development plan [2]. 

To conclude, the levels of legislation in China in relation to inclusive education are hierarchical [1]. The constitutional law did not mention inclusion directly, but its arrangement is discussed within Article 45. The National People’s Congress and its Standing Committee issued several laws that are general and specific. General laws include civil law, criminal law, and administrative law. Specific laws include the Law of Compulsory Education in 1986 on the hosting of special classes and schools and the Law of the People’s Republic of China on the Protection of Persons with Disabilities in 1990. This latter one was amended in 2008 before the ratification of the CRPD. The next level of laws included regulations from education authorities, including Chinese law on education in 1996, mainly Article 10, supporting special education. Regulations made by the State Council have less legal power, and this includes the Regulations Education for Persons with Disabilities in 1994. Further, local provinces’ people’s representatives’ congresses issue ministry regulation and local governmental regulations (e.g., The Shanghai Implementation of the Chinese Law on Protection of Persons with Disabilities. Finally, the ministries and local governments’ regulations like the National Plan 2014 to enhance special education [1].

### 1.2. Rights of Learners with Special Educational Needs

The rights of learners with Special Educational Needs (SENs) are emphasized in Chinese law, legislation, and policies. They strictly inform all education sectors and relevant sectors to ensure *equality*, *learning,* and a *sense of belonging* to all learners with SENs. For instance, the Compulsory Education Law of the People’s Republic of China, Article 19, stated two forms of providing education through special education classes (segregation) or through regular education classes (i.e., LRC) [3]. Another example is the Regulation on the Education of the Disabled details four forms of education for learners with SENs: (a) full inclusion for learners who have mild disabilities, (b) partial inclusion (mainstreaming) for learners who need minor preparation to fully join the regular education system, (c) segregation on special education schools for those who cannot match any of the previous two methods requirements, and (d) exclusion (i.e., home education) for those whose disabilities and conditions do not allow them to join schools on campus [4]. 

Historically, the government has always been highly concerned with ensuring the rights of learners with SENs. The Chinese constitution starting from 1982—revised frequently in 1988, 1993, 1999, and 2004—ensures the protection of people with disabilities. Other important laws include (a) The Law on the Protection of Disabled Persons, 1991, (b) The Rules on the Employment of Disabled Persons, 2007, (c) The Employment Promotion Law, 2007, (d) The Twelfth 5 Year National Programme on Disability (2011–2015), (e) Regulations on Construction of Accessible Environment (2012), and (f) Mental Health Law (2012) (International Labour Organization [ILO], as cited in [5].

Of particular significance, the China Disabled Persons’ Federation (CDPF) was established in 1988 to advocate for the rights and well-being of over 85 million individuals with special educational needs (SENs). As a result, numerous specialized and general associations and organizations have been established to represent this community of learners. These include the State Council Working Committee for Persons with Disabilities, China Association of the Deaf, China Association of the Blind, China Association of the Physically Handicapped, China Association of Persons with Intellectual Disabilities and their Families, China Association of Persons with Mental Disabilities and their Families (ILO) as cited in [5,6,7].

Further, major national policies emphasized ensuring equality, learning, and a sense of belonging for learners with SENs. Among these policies is the National Plan 2010–2020, which was part of the used policy documents in this study—addressing inclusion and segregation [8]. Another yet recent national policy is China Education Modernization 2035 which again emphasized the development of special and inclusive education with more focus on quality [9]. 

The results of the study might be of help to decision-makers when deciding on funding special and inclusive education especially considering bridging the gaps between policymakers and implementers. Policy-makers might also find it useful to use the output of this study, revealing the challenges faced by stakeholders with respect to inclusion and segregation. Practitioners and teachers of special and inclusive education may also find it worth increasing their awareness about the current practices, methods, and theories of special and inclusive education. To this end, the study addressed two questions:

1. Do stakeholders’ attitudes towards legislation, policy, and law regarding special education influence their roles and responsibilities?
2. In what ways do stakeholders interact with the legislation, laws, and policies of special education, and what is their work experience in the field?

## 2. Method

This qualitative, constructivist-based perspective study contributes to understanding special and inclusive education in China through the voices of stakeholders [10,11]. A major benefit of the qualitative approach relevant to this paper is that it studies the context, that is, special and inclusive education in China [12]. The remained of this section discusses the design of the study, data collection, methods, trustworthiness, ethical concerns, and data analysis. 

### 2.1. Study Participants

**Figure 1:**
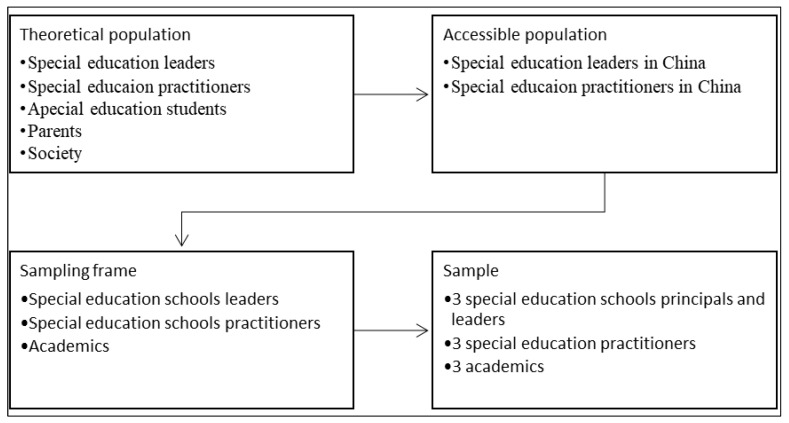
Sampling Framework.

The study follows the bioecological systems theory in which the examined topic ‘special education development’ is formed, experienced, and understood through the interactions and interrelationships among several different factors within the micro, meso, exo, macro, and chronosystems [13,14,15]. In addition to special education policymakers and practitioners, other stakeholders include special education students, parents, society, the country’s economy, and more. We do not intend to include all these factors in this study. Because of this, the researcher used non-probability sampling, which included both heterogeneity sampling for school principals and practitioners, as well as expert sampling for the 3 academic individuals included in the study. Figure 1 and Table 1 below show the sampling methodology and sociodemographic characteristics of the participants. 

### 2.2. Design

**Figure 2:**
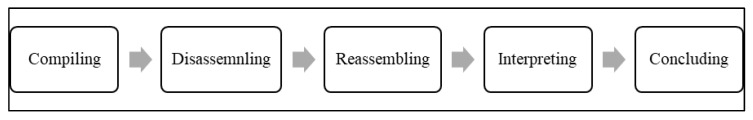
Thematic Analysis Study Design.

Nine interviews were analyzed using a thematic analysis conducted in 5 phases. It is assumed that school principals, practitioners, and academics have contributed to gaining a deeper understanding by focusing on laws and policies related to the provision of special and inclusive education services in China. Figure 2 illustrates the research design. 

### 2.3. Data Collection

Data was collected between 1 May 2019 and 30 June 2019 in Beijing, China. A total of nine interviews were conducted for this study. Within Beijing, 6 interviews took place, with 3 conducted at a special and inclusive education school and 3 at an intellectual disability school. Furthermore, 2 interviews were conducted at a university, while 1 interview was conducted online. These interviews were between 50 and 75 min long. For all interviews except the online one conducted through WeChat, in which the consent document was verbally signed, mobile phones were used to record the interviews after the consent forms were signed. While 4 academics were scheduled to participate, 1 of them subsequently refused to conduct the interview, stating that he would rather not disclose his views and experiences on the topic. 

Researchers assisting the interviewers were guided by some themes and guiding questions. The form was developed by the researcher, and some of the items and proposed questions were edited by an expert in special education. Using his comments, the prepared list was shortened, and a demographic section was added. During the interviews with nine interviewees, this expert stated only one of them knew English well enough to communicate adequately. Three researchers volunteered to conduct Chinese interviews alongside the researcher. By using WeChat, the researcher arranged and discussed the details of the interview. Some suggestions and questions were raised. A meeting was scheduled to go over their questions 1 by 1 after clarifications were provided to them and their questions answered. We also checked the procedures with 3 assisting researchers who were pursuing their master’s degrees in special education. They were advised, for example, not to discuss policy, laws, and administrative matters of special education in depth with the special education practitioners and to discuss them instead with the director, manager, and principal. In addition, they were instructed not to discuss in detail teaching methods with administrators or too deeply with special education practitioners. Nevertheless, they were told to leave this open so that all interviewees could share their experiences and views regardless of their positions. 

The supervisor approved the interviewees’ list, and the assistants helped to arrange the interview dates and locations. Each interviewee signed a consent form before beginning the interview. Researchers assisting with the interview provided them with Chinese interview questions as well. 

Following the completion of the interviews, 1 interview was first transcribed into Chinese and then translated into English. This transcript was initially deemed too long and stuffed with unnecessary details. Thus, we decided to abstract data according to the questions and themes. Research assistants had the option of transcribing directly into English or starting with Chinese and then translating into English. Nonetheless, the researcher emphasized that strict literal translation should be avoided, and their efforts should be directed toward it. The assisting researchers sent the data to the researcher via WeChat with eight Word documents. This ninth interview was conducted completely online by the researcher, recorded using a mobile phone, and directly transcribed into English. A selective transcription was also used to avoid the inclusion of unnecessary data and waste of time. 

### 2.4. Procedures 

In-depth interviews were conducted with nine special education stakeholders to collect data. As described in Yin (2018), in-depth interviews are known as “unstructured interviews” or “intensive interviews” that are “conversational in nature” (p. 351). A qualitative approach was selected to elicit more data from the interviewees and allow them to speak freely about inclusion and segregation in special education in general (Yin, 2018). The consent form and interview are included in Appendix A, Appendix B, Appendix C. According to Yin (2014), interviews can be used as a valuable tool for validating past data. As he noted, “in-depth interviews have the potential to corroborate established findings and not to expand on topics of a more general, open-ended nature” (p. 179), although gathering more data was the objective of using this approach rather than just confirming quantitative data. 

### 2.5. Trustworthiness

In the present study, we aimed to present a new understanding of special education development in China, with an emphasis on presenting a critical analysis of the conflict between policies and stakeholders. In conducting a thematic analysis for the collection and analysis of the interviews, there were five phases. As part of this, we compiled data, disassembled, reassembled, interpreted, and concluded. 

The nine interviews were analyzed primarily using content analysis [16,17]. A thematic analysis is considered trustworthy through the different phases of the process [18,19]. For this study, it was established through various methods. Credibility, transferability, dependability, and confirmability are the four main trustworthiness criteria, each of which was approached differently during each of the 5 phases of thematic analysis, including compiling, dissembling, reassembling, interpreting, and concluding. Each of these phases, the trustworthiness concepts, and the means that were used to establish them are listed in Table 2.

### 2.6. Ethical Concerns 

During the in-depth interviews, agreements were arranged at the supervisor’s end since many interviewees did not possess a good level of English, which would have allowed them to conduct the interviews in English. Before beginning the interview, all interviewees signed a consent form that was located in the Appendix B. In the ninth interview, the consent form was approved verbally, and the interview was conducted online. 

We also ensured the conformation to ethical standards during the interviews, where visited schools requested that photographs not be taken, and the names of schools and public universities were not revealed. In accordance with the interviewees’ requests, their names and affiliations were also coded. 

### 2.7. Data Analysis 

**Figure 3:**
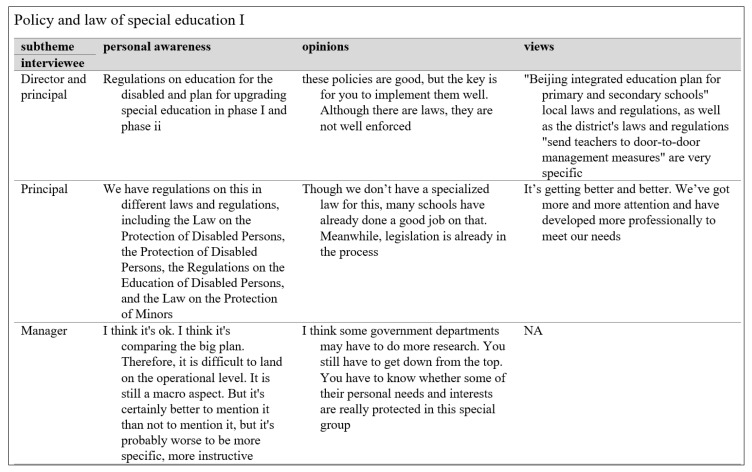
Sample Data Extraction Screenshot for Stakeholders’ Understanding and Views on Laws and Policies.

**Figure 4:**
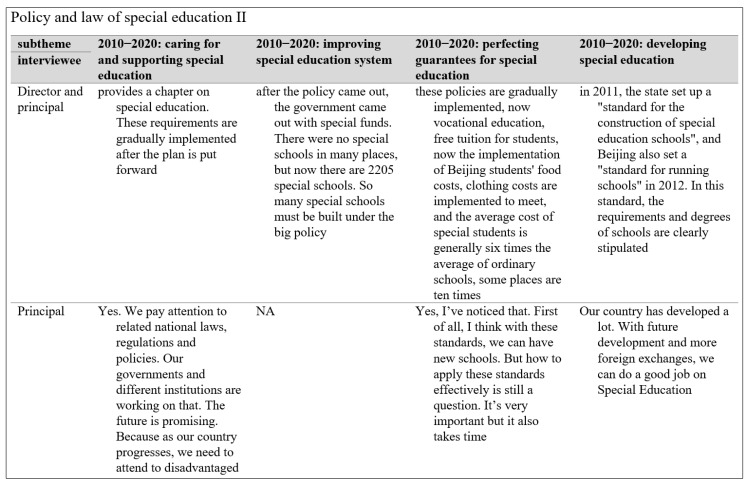
Sample Data Extraction Screenshot for Stakeholders’ Views on the National Plan 2010–2020.

The interviews were analyzed following 5 phases: compilation, disassembly, reassembly, interpretation, and conclusion (see Appendix A, Appendix B, Appendix C) for data coding, consent form and interview protocol. Once the data were compiled, disassembled, and reassembled, it was arranged based on the views and experiences of leaders, practitioners, and academics. Following the themes which were listed on the interview form, the rest of the analysis followed (see Figure 3 and Figure 4). We selected excerpts based on their relevance to the themes as well as summarizing and paraphrasing them. When discussing the results, discussing the discussion, and discussing the rest of the study, the interviewees were referred to by their titles (director, principal, manager, special education practitioner, and special education academic/expert). We replaced the names of the schools and the university affiliations of the interviewees with (special education school, intellectual disability school, university in Beijing, university in Wuhan, and university in southwest China). In instances where data corresponding to the desired theme was absent, the term “not available” (NA) was entered into the data extraction tables. 

## 3. Results 

### 3.1. Stakeholders’ Views on Policy and Law of Special Education 

#### Result Overview

While nine questions guided the interview (see Appendix C), there was also room for open discussion within these topics. Two of the questions pertain to a personal story describing the participants’ attitudes toward their motivation to work in special education. It was stated by two of the administrators that they had moved from teaching into administration versus the Director and Principal, who had 39 years of experience. Three practitioners reported their reason as the major which is similar to those academics who reported choosing special education as it was challenging and they were able to have a better job, except for the assistant professor with two years’ experience who reported being compelled to accomplish this goal.

All of the remaining questions were designed to gain an understanding of special education law and policy in China. The topic is further divided into two parts. This first part looks at awareness of special education in China and attitudes toward it. The second section discusses the National Plan 2010–2020 as it pertains to the four main instructions contained in this policy document: (1) caring for and supporting special education, (2) improving special education, (3) perfecting guarantee access to special education, and (4) developing special education. 

The analysis of the policy and law regarding special education revealed several findings. While it is true that some special education personnel may hold excessively positive attitudes towards special education policies and laws, it is crucial to emphasize the importance of developing more effective and targeted laws and policies that are specific to each province, as well as to special education and inclusive education. It is essential that these laws and policies are accurately implemented, taking into consideration the fact that the awareness of special education personnel regarding special education laws and policies is often average or below average. Additionally, it is worth noting that some special education personnel may exhibit an exaggerated negative attitude towards these policies and laws, while others may tend to over-interpret the existing laws and policies in the field of special and inclusive education.

### 3.2. Attitudes toward Inclusion and Segregation 

**Exaggerated Attitudes**. We use this concept to refer to an overemphasis or overstatement of one’s opinions or beliefs, often leading to an inflated or extreme view of a particular subject or issue. That being said, it is imperative to prioritize the development and implementation of more effective laws and policies that are tailored to the specific needs of each province, as well as to the domains of special education and inclusive education. This is crucial due to the observation that the awareness of special education personnel regarding special education laws and policies tends to be either average or below average. By issuing and accurately implementing province-specific, special education-specific, and inclusive education-specific laws and policies, we can address this gap in knowledge and ensure more effective practices in the field.

Some of the participants mentioned the below laws and policies, while some others stated that they could not recall any: 

*Regulations on the education of the disabled … law on the protection of disabled persons* *…law on the protection of minors … compulsory education law … amendment of special education policy in 2017 … National Plan 2010–2020 … disability protection…. (SES-1; IDS-1; SES-2; IDS-2; SES-3; IDS-3).*

**Negative Attitudes**. Additionally, special education personnel have exaggerated negative attitudes toward special education policies and laws. 

*Should popularize laws and policies … good policies, but need to be enforced … not specific laws but in progress … need to follow bottom-up approach … to ensure all personnel and disabled rights … have general laws, policies, and legislations … good regulations and laws but implementation is not taking place in full … lack of specific and detailed laws on special education (PU-1; PU-2; PU-3)*.

**Overinterpretations of Laws**. We use this concept to refer to the act of assigning excessive meaning, significance, or implications to legal provisions, often leading to an exaggerated or distorted understanding of their intended scope or application. Based on this, it is essential to issue and accurately implement more effective laws and policies that are tailored to specific provinces, special education, and inclusive education. This is necessary because certain individuals in the field of special and inclusive education tend to over-interpret the existing laws and policies. By developing and implementing province-specific, special education-specific, and inclusive education-specific laws and policies, we can ensure clearer guidelines and avoid misinterpretations that may hinder the effective implementation of these regulations.

*Integration laws at the province, city, and district levels in Beijing [do exist] … all are improving well … improving but still needs further policies … local and national developments are working well … remarkable progress but still needs more efforts … effective development … moving towards zero rejection (SES-1; IDS-1; SES-2; IDS-2; SES-3; IDS-3; PU-1; PU-2; PU-3).*

**Figure 5:**
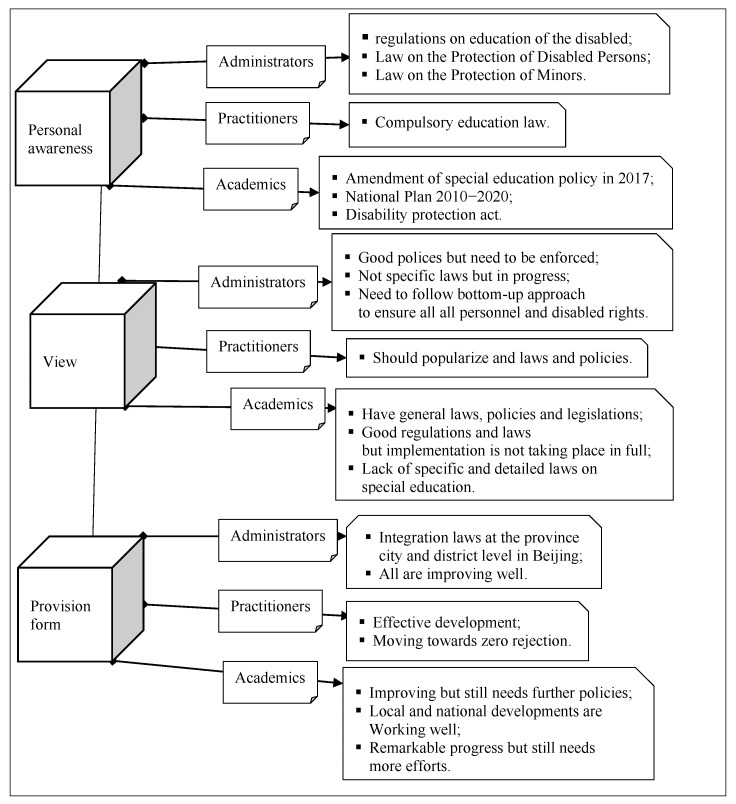
Policy and Law of Special Education I.

Hence, although special education personnel have displayed exaggerated positive attitudes towards special education policies and laws, it is crucial to develop and implement more precise laws and policies that are tailored to specific provinces, special education, and inclusive education. In this regard, Figure 5 provides a visual representation of stakeholders’ awareness, reflections, and perspectives on laws and provisions.

### 3.3. Reflections on Inclusion and Segregation in the National Plan 2010–2020 

The analysis of policy and law of special education also put more focus on the National Plan 2010–2020. Although the deadline for achieving the National Plan 2010–2020 was approaching when this study was conducted, some of the acts have not been put into effect at the national level, especially in rural areas, as evidenced by the expressed concerns of some of the participants, the National Plan 2010–2020 has been introduced to ensure both quantitative and qualitative development of special education in China because it aims at caring for and supporting special education, it considers improving the special education system, it ensures perfecting guarantees for special education, and it emphasizes developing special education. 

**Caring for and Supporting Special Education**. First, the National Plan 2010–2020 has been introduced to ensure both quantitative and qualitative development of special education in China because it aims at caring for and supporting special education where government at all levels should speed up special education development, intensive training for people with disabilities, and full integration into the society. The reflection of the participants on this particular article for reforming special education included: 

*Gradually implemented … providing personalized services representing the country’s development of economy, culture and civilization … a lot of special education policy support and some welfare support, not only for the children’s education but also for the children’s life … supervisors and managers need to learn more but not practitioners … increase in the investment including financial and policy support including teacher development and extending the 9 year compulsory education to high school and vocational education … the country’s attitude towards special education has undergone great changes, from promotion to support to success … has limited impact but a good start. (SES-1; IDS-1; SES-2; IDS-2; SES-3; IDS-3; PU-1; PU-2; PU-3).*

**Improving Special Education System**. Additionally, it considers improving the special education system proposing that a county with over 300,000 with disabilities should have a special education school; schools create opportunities to accept them in regular education and preschool education, senior middle school, vocational education, and higher education are accelerated. Some of the interviewees showed total agreement that these are clearly observed, and they declared: 

*There were no special schools in many places, but now there are 2205 special schools … in the past 5 or 6 years, the Haidian district has paid more and more attention to integrated education … more and more special education schools are opening; before 2010, very few schools were providing special education for vocational education (SES-1; IDS-1; SES-2; IDS-2; SES-3; IDS-3; PU-1; PU-2; PU-3).*

Nevertheless, some others think that the realization of this Article is still partial and claim: 

*Although there are regulations in the policies, it is difficult for kindergartens to accept special education children … the policy of setting up schools based on population is not fair … high school, vocational education and higher education still need too much to be done. (SES-1; IDS-1; SES-2; IDS-2; SES-3; IDS-3; PU-1; PU-2; PU-3).*

**Perfecting Guarantees for Special Education**. Furthermore, it ensures perfecting guarantees for special education by making basic national standards for the operation of special education set by the local government, more investment, ensuring that regular schools provide a suitable environment, increasing the salaries of special education teachers, funding special education in the community (hard situation families), and providing free senior middle school gradually. The views on this Article are mixed between crediting its full realization and the need to issue further detailed policies and laws to ensure the best special education services. 

*In the implementation of Beijing students’ food costs, clothing costs are gradually met … this is being noticed but still needs to be more effective … preschool education and vocational education related to special children, especially those with mental retardation, are very weak … in some areas, they do not even have a high school within their system … the basic salary for them should be 30% more than regular teachers … more policy documents need to be issued in the next 10 years … the gap between regions, unbalanced investment and spending, and the economic status of families need urgent and further reform. (SES-1; IDS-1; SES-2; IDS-2; SES-3; IDS-3; PU-1; PU-2; PU-3).*

**Comprehensive Development**. Finally, the National Plan 2010–2020 has been introduced to ensure both quantitative and qualitative development of special education in China because it emphasizes developing special education where schools are refurbished, expanded, or new ones built, education conditions should have necessary teaching, living, rehabilitation training facilities, and teachers have professional training. Positive views included: 

*The standards for schools’ construction were set in 2011, and in Beijing in 2012 … with more development and more foreign exchanges, we can do better … hoping to see further development in teacher training … inequalities among different areas … … too many professional training programs for special education teachers, including those in schools, districts, and provinces (SES-1; IDS-1; SES-2; IDS-2; SES-3; IDS-3; PU-1; PU-2; PU-3).*

Dissimilar to such positive reflections, some others think: 

*Lots of schools in poor areas are rebuilt, and some are refurbished so that teachers can have more access … within regions such as Beijing, there are a lot of schools that have different levels of development, education is far worse, and nationally, the imbalance is bigger (SES-1; IDS-1; SES-2; IDS-2; SES-3; IDS-3; PU-1; PU-2; PU-3).*

**Figure 6:**
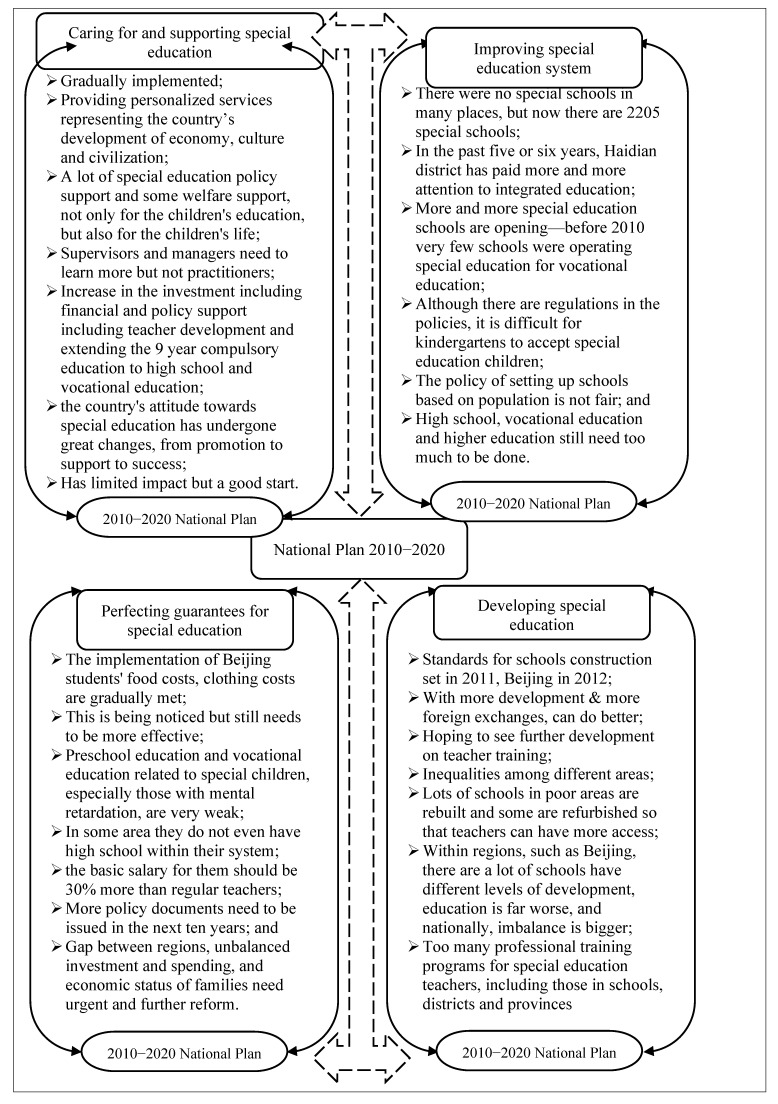
Policy and Law of Special Education II.

Hence, while the deadline for achieving the National Plan 2010–2020 was approaching when this study was conducted, some of the acts have not been put into effect at the national level, especially in rural areas, as evidenced by the expressed concerns of some of the participants, the National Plan 2010–2020 has been introduced to ensure both quantitative and qualitative development of special education in China. Figure 6 below is a visualization of the stakeholders’ understanding and views on the National Plan 2010–2020 Articles related to special and inclusive education. 

## 4. Discussion

The purpose of this study was to uncover how Chinese stakeholders perceive laws and policies regarding special education and inclusive education throughout the country. These findings reveal the possible gaps between policy-makers and implementers (i.e., stakeholders) and how this gap may affect the system’s outcome. The findings may be broken into two parts. In the first part, the following findings were found: (1) academics and administrators have a deeper understanding of and familiarity with laws and policies than practitioners; (2) the included stakeholders have exaggerated views and interpretations of the current laws and policies; and (3) while some of the included stakeholders think the provision is moving forward despite a few shortcomings, others believe that development should move to a bottom-up approach and more specific policies and laws should be issued to developing and underdeveloped areas.

The second section of the findings includes the following: (4) in regard to promoting special education, it appears that the government has been shifting from promotion to real support and achievement, although this remains limited in rural areas, (5) when it comes to improving special education, there are also some signs of progress between 2010 and 2020. However, special education services at the high school, vocational school, and preschool levels remain insufficient, (6) For the development of special education standards, it is believed that these standards move from the top to the bottom, which takes some time to reach the remote areas in a large country such as China, and (7) for perfecting the special education system, they believe this is focused on major provinces and cities such as Beijing and Shanghai, and there is no investment in other regions across the country.

It is clear from these findings that China has made significant progress in its special education system, but there are still several issues and issues that need to be addressed to provide better services for the Special Education community [20,21,22]. There have also been reports of the narrow definition of special education in China [23], the gap between rural and urban areas [24], social justice and special education services [25], social support for teachers [26], and curriculum relevant to specific categories of this community [27].

Two interpretations are possible based on these findings. It is apparent from the understanding and reflections of the included stakeholders that there are exaggerated attitudes toward the development of special and inclusive education in China. Compared to the rest of the world, these views included either negative attitudes asserting development is slow and restricted to large cities and provinces or positive attitudes asserting that the current situation represents the best of the available options. There is a possibility that these views can be attributed to an oversimplification of laws and policies as well as nationalist views. But what is actually evident is that there is significant progress and a successful special education system across the country regardless of the gaps in the different regions.

The National Plan for the Development of Education, which includes special education, has had a profound effect on the quality and quantity of services provided across the country. Evidence included opposing views that this development occurred only in basic and middle schools but not in preschool, high school, vocational schools, or universities. 

Several implications can be drawn from the above interpretation. First, the understanding of laws and policies by stakeholders influence their interaction with their working environment (e.g., schools, learners, society) and, as a result, either facilitates or hinders the performance of their work. The stakeholders within our study demonstrated high levels of enthusiasm and positive attitudes toward the practicality of the policies and laws being issued and implemented in China. There is no doubt that they express some negative views regarding the discrepancy between regions, and they place greater emphasis on certain levels (basic schools and middle schools) over others (i.e., preschools, high schools, vocational schools, and higher education).

As a result, it has been confirmed that the National Plan 2010–2020 has been successful, indicating that all stakeholders and, hopefully, the special education community, parents, and society as a whole, are aware of the enhancement of services provided in the fields of special education and inclusive education. While this affirmation includes several limitations on such policies, these limitations should serve to show the government why it should switch to a bottom-up approach (i.e., more budget and spending for underdeveloped or developing regions) rather than a top-down approach.

The findings and interpretations of this study have significant implications for student wellbeing and mental health. Ensuring that special education services are comprehensive, inclusive, and accessible to all students, regardless of their geographical location or level of education, is crucial for fostering a supportive environment that promotes positive mental health outcomes for students with diverse learning needs.

Addressing the gaps in understanding and implementation of laws and policies among stakeholders can lead to a more equitable and effective special education system, ultimately benefiting students’ wellbeing. By enhancing the quality and accessibility of special education services across various regions and educational levels, students with special needs will receive the necessary support for their mental and emotional well-being. Furthermore, a bottom-up approach that emphasizes investment in underdeveloped or developing regions can lead to more equitable access to services, reducing the disparities in mental health support for students with special needs. This approach acknowledges the importance of a comprehensive and cohesive special education system in promoting social justice and fostering a sense of belonging and wellbeing for all students, regardless of their unique learning needs.

Lastly, fostering open communication and collaboration among all stakeholders involved in special education and inclusive education can have a positive impact on student wellbeing and mental health. When administrators, educators, parents, and policymakers work together to better understand and implement laws and policies, they create an environment that is more supportive and responsive to the unique needs of students with disabilities. This collaborative approach helps ensure that special education services are tailored to students’ individual needs, promoting their overall wellbeing and mental health. Furthermore, increased awareness and understanding of the challenges faced by students with special needs can contribute to a more empathetic and inclusive educational environment, further enhancing their mental health and sense of belonging within their school communities.

## 5. Limitations

The present study aimed to reveal a more in-depth understanding of Chinese stakeholders regarding the laws and policies regarding special and inclusive education. There are, however, two limitations in the study. Firstly, the collected data was limited to three categories of stakeholders (administrators, academics, and practitioners). Due to linguistic and administrative barriers, it was not possible to gain access to other stakeholders. Secondly, the stakeholders involved are mainly from developed areas in China, and their views may fall in line with the developed environment in which they work. 

Another potential limitation of the study is the relatively small sample size, consisting of only 9 interviews. However, it is important to note that these interviews were conducted in-depth, allowing for rich and detailed insights from participants. Despite the limited number, the depth of the interviews can provide valuable information for understanding stakeholders’ perspectives.

Furthermore, it should be acknowledged that the majority of the interviews were conducted in Beijing, which is the location of the central government and known for its advanced development. This geographic concentration may introduce some bias in the data, as it may not fully capture the experiences and perspectives of stakeholders in underdeveloped or developing regions across the vast country of China. It is important to consider the potential limitations of generalizing the findings to the entire country.

We acknowledge these limitations and their potential impact on the generalizability of our findings. Future studies could aim to include a larger and more diverse sample, incorporating perspectives from stakeholders across various regions in China to provide a more comprehensive understanding of the issues related to special education and inclusive education legislation, law, and policy.

## 6. Conclusions 

In this study, we sought to explore Chinese stakeholders’ understandings and experiences of special education policies and laws. In our findings, the attitudes of stakeholders and their interaction with policy play a significant role in the effectiveness of their work and in the implementation of policy. Participants showed both positive and negative attitudes toward certain laws and policies. The major concerns are the gaps in development, budget allocations, and reforms across the country. Although national policies are issued, their implementation is left up to each school, city, and province, and this is where gaps emerge, whether they arise with regard to policies and their implementation or service provision across the country or even between one school and another within a city.

## Figures and Tables

**Table 1:** Sociodemographic Characteristics of the Participants.

Position	Age	Gender	Experience	Major	Location	Institution	Pseudonym	Code
Director and principal	60	Male	39	Teacher Education	Beijing	Special Education School	A special education school director	SES-1
Principal	52	Female	31	Special Education	Beijing	Intellectual Disability School	A special education school principal	IDS-1
Manager	35	Female	14	Special Education	Beijing	Special Education School	A special education school manager	SES-2
Practitioner	38	Male	16	Special Education	Beijing	Intellectual Disability School	A special education school practitioner	IDS-2
Practitioner	33	Female	7	Special Education	Beijing	Special Education School	A special education school practitioner	SES-3
Practitioner	30	Female	3	Primary Education	Beijing	Intellectual Disability School	A special education school practitioner	IDS-3
Assistant Professor	33	Male	11	Special Education	Wuhan	Public university	An academic	PU-1
Postdoctoral fellow	29	Male	7	Special Education	Beijing	Public university	An academic	PU-2
Assistant Professor	30	Male	2	Special Education	Chongqing	Public university	An academic	PU-3

**Table 2:** Trustworthiness Establishment at the Five Phases of Thematic Analysis.

Phase	Concepts	Means	Explanation
Compiling	Credibility	Triangulation	The study used in-depth interviews; however, these interviews were conducted under the guidance of some guiding themes and questions that enabled the verification of the collected data from nine interviews.
	Transferability	Thick description	The study presents a detailed description of data compilation in its methods section.
	Dependability	Detailed documentation	This study provides detailed instructions on how to compile data from the procedure section.
	Confirmability	Peer checking	Researchers in special education verified the data and, further, the researcher himself verified the data.
Disassembling	Credibility	Triangulation	An in-depth interview generated a large amount of data. It took a long time to transcript the first interview, which led to thick data. Consequently, the data was selectively transcribed. The process of transcribing everything and then translating everything into Chinese was incredibly time-consuming. To perform the first step of verification for the abstracted data, we selected one of the three researchers who were best at English.
	Transferability	Thick description	An in-depth description of coding and analysis can be found in the procedure and data analysis section.
	Dependability	Detailed documentation	A complete description of the disassembling process was provided.
	Confirmability	Peer checking	As part of the three researchers helping with the interview, transcription, and translation into English, one of them also checked the coding of themes and analysis steps.
Reassembling	Credibility	The emic or folk perspectives of the participants	Researchers who contributed to data collection were instructed to avoid any changes to the questions and to avoid leading the interviews in any direction. In addition, they were informed to give the interviewees full freedom to share their opinions and experiences without pressuring them toward the interviewers’ or researchers’ views. Moreover, the researcher requested that researchers transfer the data honestly and literally and avoid creative translation by all means.
	Transferability	Thick description	A detailed description of the final themes is provided on the data analysis page.
	Dependability	Detailed documentation	A detailed description of all steps, processes, and procedures is provided.
	Confirmability	Peer checking	For the purposes of ensuring that the final data is not beyond the scope of the study, both the generated and emerging themes were compared to the study’s objectives. The comparison was verified by a peer in the same field.
Interpreting	Credibility	Progressive subjectivity checks	Occasionally, the researcher checked if the data analyzed supported some proposed theses or contradicted others. This has been left and further discussed in the study discussion, limitations, and future research.
	Transferability	Thick description	A detailed explanation of the interpretation is provided in the procedure section and data analysis section.
	Dependability	Detailed documentation	The steps, processes, and procedures of interpretation are documented in detail.
	Confirmability	Repeated checking	The interpretation is then verified and confirmed through repeated checks.
Concluding	Credibility	peer debriefing	Peers suggested going straight to the points with the conclusions and their relevance and value to the field, society, and country.
	Transferability	Thick description	For results, analysis and interpretation were used. For conclusions, discussion and inference were used.
	Dependability	Detailed documentation	Detailed documentation is available on all steps, processes, and procedures involved in concluding data.
	Confirmability	Peer checking	The conclusions were peer-reviewed and are based on the findings and yet still answer the research questions.

## Data Availability

The data presented in this study are available on request from the first author.

## Appendix A

Policy and law of special education I

**Subtheme**	**Personal Awareness**	**Opinions**	**Views**
**Interviewee**			
Director and principal	Regulations on education for the disabled and plan for upgrading special education in phase I and phase ii	these policies are good, but the key is for you to implement them well. Although there are laws, they are not well enforced	“Beijing integrated education plan for primary and secondary schools” local laws and regulations, as well as the district’s laws and regulations “send teachers to door-to-door management measures” are very specific
Principal	We have regulations on this in different laws and regulations, including the Law on the Protection of Disabled Persons, the Protection of Disabled Persons, the Regulations on the Education of Disabled Persons, and the Law on the Protection of Minors	Though we don’t have a specialized law for this, many schools have already done a good job on that. Meanwhile, legislation is already in the process	It’s getting better and better. We’ve got more and more attention and have developed more professionally to meet our needs
Manager	I think it’s ok. I think it’s comparing the big plan. Therefore, it is difficult to land on the operational level. It is still a macro aspect. But it’s certainly better to mention it than not to mention it, but it’s probably worse to be more specific, more instructive	I think some government departments may have to do more research. You still have to get down from the top. You have to know whether some of their personal needs and interests are really protected in this special group	NA
Practitioner	“compulsory education law” “minor protection law”; be compulsory education to want to protect the right that every minor enters a school, include special children, if accord with policy, cannot reject them.	Don’t understand	China’s special education policy is constantly improving. In the whole development of special education, new problems and new demands will appear constantly, which requires constant adjustment and supplement of policies. For example, the government has been quite tough in recent years. There used to be rejection, but now as long as the child is in line with the policy, they have to accept.
Practitioner	94–142 public law. Or something with little restrictions from the environment. I don’t know anything else about it.	We should step up popularization. Based on that, people will become more aware and it’s better for implementation. On the other hand, as workers in special education area, we should learn about these laws and regulations as well.	We should step up popularization. Based on that, people will become more aware and it’s better for implementation. On the other hand, as workers in special education area, we should learn about these laws and regulations as well.
Practitioner	NA	NA	NA
Assistant Professor	I immediately think of the special education policy for students with special educational needs which was amended in 2017. I think there is also another of the national law for people with disabilities, the protection of people with disabilities.	I think we do not have any specific education law and legislation but I think it is very general and it does not stipulate all details that are not flexible.	The special education policy in China, we started to put forward lots of policies, but we still need more policies.
Postdoctoral fellow	Outline of national medium and long-term education reform and development plan 2010–2020, which is a development plan for the development of special education in recent ten years.	all the things stipulated by law are good. The contents stipulated in the education regulations for the disabled in 1993 are very good, but they have not been implemented. Many regulations will not be held liable if someone violates them.	generally speaking, the country’s improvement in special education is from various aspects. These two promotion plans, from the national to the local to promote the development of special education in many ways, and the policy enables schools, including the education department, to follow the policy and follow the rules in the implementation.
Assistant Professor	well, one is the disability protection act of recent years, and then there are some policies and regulations that we usually talk about, to upgrade the program. There is also, the course that those who set before, disabled course standard. The standard of course teaching respect, next of course big scope is a few education law, these of compulsory education law.	so far our country special education related laws he is not, is that many law is mainly in other laws, such as education, security regulations for the disabled, he has some Settings, but involves the aspects of persons with disabilities act, it is a big, is there is a part of the content of the education, so the special education in our country related laws is very lack, or there is no specific law.	generally speaking, our country’s special education policy in the new century, or 2010 years later, the whole policy of special education is more and more perfect, began to gradually, so more and more its policy, the policy mainly includes some policies formulated by the national policies of national level, then of course including some developed areas or other areas, so according to the requirement of the country, many policies, on the other hand is the policy of our country, special education policy, or far cannot satisfy the now, so the need of special education.

Policy and law of special education II

**Subtheme**	**2010−2020: Caring for and Supporting Special Education**	**2010−2020: Improving Special Education System**	**2010−2020: Perfecting Guarantees for Special Education**	**2010−2020: Developing Special Education**
**Interviewee**				
Director and principal	provides a chapter on special education. These requirements are gradually implemented after the plan is put forward	after the policy came out, the government came out with special funds. There were no special schools in many places, but now there are 2205 special schools. So many special schools must be built under the big policy	these policies are gradually implemented, now vocational education, free tuition for students, now the implementation of Beijing students’ food costs, clothing costs are implemented to meet, and the average cost of special students is generally six times the average of ordinary schools, some places are ten times	in 2011, the state set up a “standard for the construction of special education schools”, and Beijing also set a “standard for running schools” in 2012. In this standard, the requirements and degrees of schools are clearly stipulated
Principal	Yes. We pay attention to related national laws, regulations and policies. Our governments and different institutions are working on that. The future is promising. Because as our country progresses, we need to attend to disadvantaged people and provide personalized services for them, because that represents a country’s development of economy, culture and civilization. Now China has valued civilization and environmental harmony. In this way, Special Education can have a bright future. Now, I think we need to build a support system for Special Education. Integrated education requires us to respect people. So need a system in which special education schools are not just places for these students, but also places of research. Special education schools need time to develop	NA	Yes, I’ve noticed that. First of all, I think with these standards, we can have new schools. But how to apply these standards effectively is still a question. It’s very important but it also takes time	Our country has developed a lot. With future development and more foreign exchanges, we can do a good job on Special Education
Manager	NA	NA	NA	NA
Practitioner	the medium- and long-term plan will be realized next year. This policy is to give parents a great hope, parents are very concerned about this policy. Because there are a lot of special education policy support and some welfare support, not only for the children’s education stage, but also for the children’s life stage.	in the past five or six years, haidian district has paid more and more attention to integrated education. At the very beginning, the special education center was set up by our school. Later, the special education center became independent and took charge of integrated education of the whole district. This is particularly well done. Although I am a special education teacher, I still hope that children can live in a normal environment and contact with other children. Otherwise, it is a relatively closed environment, and the people and things contacted are very narrow.	I think we have done a good job in education, but we still need to strengthen compulsory education. For example, preschool education and vocational education related to special children, especially those with mental retardation, are very weak.	I know, later schools is a standard, need to build specific I don’t know what kind of school, and then, this piece, education conditions improved over the years is really great, as long as it is the school’s side have the development needs, countries can offer certain support, and then, the teacher training efforts in this piece is big, if schools have a what kind of training needs, can have the funds to do support, because it is rich in resources, on this side of the Beijing will find corresponding resources for training.
Practitioner	When they just get rolled out, we should learn about it and have a basic understanding of it. Supervisors and managers need to learn more. Practitioners should have a basic understanding. But I think frontline teachers don’t need to spend much time analyzing them.	NA	On one hand, it’s learning in the field. On the other hand, we communicate with friends.	In this way, there might be inequalities among different areas. So we need to find a balance.
Practitioner	NA	NA	NA	NA
Assistant Professor	I think it is happening now because the outline is a guiding policy but in 2014 a number of ministries and federation put forward the improvement plan and also in 2017 which referred to this outline too. They mentioned we should increase the investment including the financial support and policy support including teacher development and extend the 9 year compulsory education to high school and vocational education. I think it is happening in regard to the word caring and support of special education. More and more special education schools are opening. Before 2010 very few schools were operating special education for vocational education. It depends on the different areas and different school. For example Shenzhen, they are very rich and they have so much support. In other settings, they may need to pay part of it or all.	More and more special education schools are opening. Before 2010 very few schools were operating special education for vocational education. It depends on the different areas and different school. For example Shenzhen, they are very rich and they have so much support. In other settings, they may need to pay part of it or all.	It is relative. In some area they do not even have high school within their system. I am not sure about salaries but I know the basic salary for them should be 30% more than regular teachers.	I think all these thing are happening based on my observations. I can see lots of schools in poor areas which are rebuilt and some are refurbished so that teachers can more access.
Postdoctoral fellow	in the last ten years, the country’s attitude towards special education has undergone great changes, from promotion to support to success. Although it has only changed two words, it can be found that the country attaches more and more importance to special education.	it is mainly the development of preschool education and higher education. The country mainly pays attention to the stage of compulsory education and pays little attention to the development of both ends. The present system is still large in the middle and small at both ends. For preschool education itself, many policies concerning preschool education stipulate special education, but although there are regulations in the policies, it is difficult for kindergartens to accept special children. Due to the lack of preschool education resources, it is difficult to implement in practice. Unless the preschool education into the compulsory education stage.	policy documents need to be carried out continuously, especially from 2010 to 2020, and more policy documents need to be issued in the next ten years. In my opinion, new positioning is needed in the future. What exactly should special education do? These questions need to be clarified.	the first is the operability of the policy, must be the implementation of the policy. The balance of the second is the area is very important, from the development of special education, and there is no any resources followed in the countryside, but in the countryside, many of the students without disabilities appraisal, followed in the while, followed in the but no money, the gap leads to the region, within the region, such as Beijing, there are a lot of schools have different levels of development, education is far far worse, and nationally, imbalance is bigger.In addition, in the field of special education, the development of informatization is of great help to the development of special education in terms of teachers’ professional quality and the concept of curriculum implementation. Many information technologies are mainly used in rehabilitation, but not in curriculum teaching. Therefore, how to better use the Internet is a problem worth thinking about in the future.
Assistant Professor	well, of course I pay attention to him for that. But I feel the whole plan, well, on the one hand, the development of the whole person education as a guide, it is now common education, teachers, especially the leaders, this aspect of consciousness gradually improve, but on the other hand, because there is too much it involves a plan, then the content of the special education as one of the small. Its impact is still very limited. Well, but it should be a good start	there are more than 300,000 disabled line, should that is special education school, so now, more than 300,000 people in the middle and western regions of the county, and many have set up special education school, but also these problems, is below 300,000 population, he how to build special education school, or how the children placed, so although it is said to inclusive into regular school education, but for the Midwest, 300,000 population below special man some county, for example of my county, so he has no special education school, and many people with disabilities is difficult to go to school.Second is school should create the opportunity to receive him, formal education is just mentioned, the central region in the eastern areas or coastal regions, we now no matter how much you are more than 300,000 or above, then these special children, more and more into the regular school accept education, but this is hard to realize in the Midwest, is the whole word for the disabled or the Midwest and rural areas of still higher unemploymentThird, to speed up the pre-school education, high school, professional education and higher education, special education, it should be a system because so far many special children is 9 years of compulsory education after graduation may be faced with the home state, or some words or after 9 years of compulsory education, still stay in school to 20 years old, and even some of the children, I also saw more than 20 years old before all stay in the school, so the whole is our, professional education a piece of this a school or higher education school, is to continue to grow, and how to make these children accept special education and culture after nine years of compulsory education,	the country basic national standards for special education work, or I think this is the most need is urgently needed to solve the problem, because in our country’s education policy and school on the whole, many measures, it often according to policy of the country, if countries do not have very good policy, or does not have special policy for this so, when the practice of the following may be compromised, especially some, still underdeveloped areas, it does not have the resources, it may be compromised.Second standards formulated by the government of the place, for the eastern region or some developed urban areas, then gradually have their own standard, then explore its own pattern, I think this pattern is very important, because if for the education of special education in China, a lot of differences in these, so each region according to their own, their needs and their actual practice the standard is very important.The third is the more investment, so this is one of the most important of course, by mentioning how to education, are now facing a lot of it is school recruitment of students after the special children, he didn’t have enough teachers don’t have enough power, so to protect the rights of these children, as well as special children, no more teachers, resources protection without causing a normal child.There is a special education is funded community and families, I think this one is also very important, is the whole, there are many disabled children family, one is economic pressure, another is, how to through the community education, community school so that all parents can rest assured to work, because once the disabled children is now close to home, and then facing unemployment for a family with a staff, so in this case, he may have certain benefits to the child, but the economic burden of the whole family will be more and more heavy, so how about funding community family, so to subsidise the community,	Or renovation and expansion of new answer: school, so now the situation is more and more, is the whole school renovation of expansion or more and more new schools, so this is developing quickly, the second necessary teaching life rehabilitation training facilities, then this, is the national investment in recent years, is also is a large part, this is rehabilitation facilities, some of the basic life need, is basically a special education school, can basically meet the needs of special education students. As for the third teacher professional training, there are too many professional training programs for special education teachers, including those in schools, districts and provinces. The training organized in the city and the training organized in the province, so, this training is more and more, basically can meet its current needs.

## Appendix B

Interview Consent Form

**Research project title**: Special education development in China between inclusion and segregation: Insights from the bio-ecological systems theory

**Research investigator:** Ahmed Alduais

**Research Participants name/position/job:**

The interview will take around (**1 h**). We don’t anticipate any risks associated with your participation, but you have the right to stop the interview or withdraw from the research at any time.

Thank you for agreeing to be interviewed as part of the above research project. Would you, therefore, read the accompanying **information sheet** and then sign this form to certify that you approve the following:

- the interview will be recorded, and a transcript will be produced
- the transcript of the interview will be analysed by (Ahmed Alduais) as a research investigator
- access to the interview transcript will be limited to (Ahmed Alduais) and academic colleagues and researchers with whom he might collaborate as part of the research process
- any summary interview content, or direct quotations from the interview, that are made available through academic publication or other academic outlets will be anonymized so that you cannot be identified, and care will be taken to ensure that other information in the interview that could identify yourself is not revealed
- the actual recording will be (destroyed after the research is over)
- any variation of the conditions above will only occur with your further explicit approval

### Quotation Agreement

**I also understand that my words may be quoted directly. With regards to being quoted, please initial next to any of the statements that you agree with:**

I wish to review the notes, transcripts, or other data collected during the research pertaining to my participation.
I agree to be quoted directly.
I agree to be quoted directly if my name is not published and a made-up name (pseudonym) is used.
I agree that the researchers may publish documents that contain quotations by me.

All or part of the content of your interview may be used;

- In academic papers, policy papers, or news articles
- On our website and in other media that we may produce, such as spoken presentations
- On other feedback events
- In an archive of the project as noted above

By signing this form, I agree that;

1. I am voluntarily taking part in this project. I understand that I don’t have to take part, and I can stop the interview at any time;
2. The transcribed interview or extracts from it may be used as described above;
3. I have read the Information sheet;
4. I don’t expect to receive any benefit or payment for my participation;
5. I can request a copy of the transcript of my interview and may make edits I feel necessary to ensure the effectiveness of any agreement made about confidentiality;
6. I have been able to ask any questions I might have, and I understand that I am free to contact the researcher with any questions I may have in the future.

### Contact Information

This research has been reviewed and approved by the Faculty of Education, Beijing Normal University, as part of the PhD dissertation in Comparative Education. If you have any further questions or concerns about this study, please contact:

Name of researcher Full address: Ahmed Alduais

Tel.:

E-mail: ibnalduais@gmail.com

You can also contact: Professor DENG, Meng

Full address: Institute of Special Education, Faculty of Education, BNU

Tel.: +86-180-46-503-188

E-mail: mdeng@bnu.edu.cn

** Note: this form is adapted in part and/or in whole from the interview consent form of the University of Edinburgh, School of Geosciences, UK.*

## Appendix C. Interview Guideline

Interviewees

**Type**	**Number**
special education experts	3
special education practitioners/teachers	3 (inclusive and special *educations*)
school managers/leaders	3 (mixed schools)
**Total**	9

**Interview pre-determined questions/themes**

**Demographics**

Name:

Gender:

Age:

Years of Experience:

Major:

School Location:

**Topic 1: Personal Story**

1. Could you tell me why did you choose to work on special education?
2. How long have you been working on education in general and special education in particular? And what have you been doing exactly?

**Topic 2: Special Education Law/Policy**

3. Generally speaking, what kind of laws and policies can you recall for special education?
4. What is your opinion on special education law in China?
5. How do you think about special education policy in China?
6. The National Plan 2010−2020 mentioned caring for and supporting special education: as a special education expert/practitioner, have you been observing this in reality? How do you think about it?
7. The National Plan 2010−2020 mentioned improving special education system: as a special education expert/practitioner, have you been observing this in reality? How do you think about it?
  - County with over 300,000 with disabilities should have a special education school
  - Schools should create opportunities to accept them in regular education
  - Preschool education, senior middles school, vocational education and higher education should be accelerated
8. The National Plan 2010−2020 mentioned perfecting guarantees for special education: as a special education expert/practitioner, have you been observing this in reality? How do you think about it?
  - State make basic national standards for operation of special education
  - Local government sets their own standards
  - More investment
  - Regular schools should provide suitable environment
  - Salaries of special education teachers
  - Funding special education community (hard situation families)
  - Free senior middle school gradually
9. The National Plan 2010−2020 mentioned developing special education: as a special education expert/practitioner, have you been observing this in reality? How do you think about it?
  - Schools: refurbished, expanded, or new ones built
  - Education conditions: necessary teaching, living, rehabilitation training facilities
  - Teachers: professional training
